# Generic substitution for prescribed brand medicines in Ethiopia: knowledge, attitude and practice among pharmacy professionals in community drug retail outlets

**DOI:** 10.1186/s12913-022-08330-6

**Published:** 2022-07-19

**Authors:** Sintayehu Alemu, Natnael Tadesse, Tidenek Mulugeta, Desta Assefa

**Affiliations:** School of Pharmacy, Institute of Health, Jimma University, Jimma, Ethiopia

**Keywords:** Brand medicines, Generic medicines, Generic substitution, Jimma town

## Abstract

**Background:**

Generic substitution is a good approach to reduce pharmaceutical expenses without compromising healthcare quality. Yet, the practice of generic substitution has been contentious due to concerns on quality and efficacy.

**Objective:**

This study was aimed to assess knowledge, attitude and practice among pharmacy professionals toward generic substitution in community drug retail outlets in Jimma town, Southwest Ethiopia.

**Methods:**

A descriptive cross-sectional study was conducted among pharmacy professionals working in community drug retail outlets. Data was collected using a self-administered questionnaire. Knowledge was tested using a 3-point response format consisting of “Yes,” “No” and “I am not sure.” Attitude was evaluated using the 5-point Likert scale ranging from 1(strong agreement) to 5 (strong disagreement). The practice was examined as never, seldom, sometimes, often, and always with scores ranging from 0 to 4. The influences of socio-demographic factors on knowledge, attitude, and practice were tested using the Mann–Whitney U and Kruskal–Wallis tests as appropriate. *P* ≤ 0.05 was considered statistically significant.

**Results:**

The mean knowledge score of participants regarding generic medicines was 5.75 ± 1.79. Only 32 respondents (30.2%) of the participants were knowledgeable about the generic substitution. 54 (50.9%) of respondents had positive attitude toward generic substitution and 52 (49.1%) had practiced generic substitution. The year of experience had a significant effect on knowledge (X^2^ = 9.14, *p* = 0.01) and practice (X^2^ = 4.71, *p* = 0.03) of generic substitution.

**Conclusions:**

Our study found that pharmacy professionals working in community drug retail outlets in Jimma town had lack of knowledge about generic substitution. Conversely, an enormous amount of participants had positive attitude toward generic substitution and nearly half of them had practiced generic substitution. The year of experience had a significant effect on knowledge and practice of generic substitution.

## Introduction

The increase in healthcare costs could be a worldwide concern within which drug expenses represent a major percentage of those costs [1–3]. Thus, governments in many countries have adopted ongoing series of cost-containment attempts in an endeavor to provide equitable access to healthcare. One among the numerous ways to regulate healthcare expenditure is to push the employment of cheaper generic medicines rather than costlier branded equivalents [4–7].

Generic medicines have the same active ingredient, strength, route of administration, safety, dosage form, efficacy, quality, intended use, and bioequivalent to brand medicines. Thus, they are usually intended to be interchangeable with (substitute for) the branded medicine [8–11]. Generic medicines are sold at a far lower cost. They are typically 20 to 90% cheaper than their brand equivalent. Hence, generic medicines are cheap alternative to costlier branded medicines without compromising healthcare quality [8–10].

Generic substitution (GS) refers to a substitution of a prescribed brand medicine by a different form of an identical active substance or switching from prescribed brand medicines to identical generic medicines [6, 12]. The practice of GS is strongly supported by health authorities globally [13]. GS can improve medicines utilization by making it more accessible and affordable [1]. However, the practice of generic medicines substitution has been controversial among healthcare professionals, particularly due to issues on quality, safety, and efficacy. Generics medicines are perceived as of low quality, less effective and less safe than brand medicines [14, 15].

Indeed, the misconceptions concerning quality, efficacy and safety on generic medicines need to be corrected to promote GS [4]. Wrong or suboptimal knowledge and attitude about generics becomes a major hindrance in a wider GS [13]. In this regard, the knowledge and attitude of pharmacy professionals is pivotal in abolishing the argument with GS and encourage practice of GS. In our study context, pharmacy professionals include both pharmacists and druggists. Given the role of pharmacy professionals in the management of medicines, including the selection and dispensing of medicines, they could be an important point of intervention for promoting generic medicine utilization in the health care system. This is dependent on knowledge and perception of pharmacy professionals. Therefore, it is vital to evaluate the knowledge of pharmacy professionals on generic medicines and their attitude and practice toward GS which is a precondition to inspire its utilization.

Both governmental and private institutions train students in pharmaceutical education in Ethiopia right now [16]. The institutions train druggists with diploma program and pharmacists in degree programs. Professional’s knowledge and attitude is closely linked with the instructional quality of training institutions. In this regard, the Higher Education and Relevance Quality Agency (HERQA) is responsible to oversee the standard of instruction delivered in Ethiopian higher education. The concept of quality culture is not well developed in Ethiopia, and as a result, there is no well-established quality assurance system. Varieties of stakeholders need to play a part in the defining of methods and criteria for external quality assurance. Despite this, HERQA is the only stakeholder group with a formal role [17].

In Ethiopia, the national drug policy offers pharmacists the right to dispense generic medicines as substitutes for prescribed brand medicines [9, 13]. There are few studies on knowledge, attitude and practice of pharmacy professionals on generic medicines importance in Ethiopian [9, 13, 18–20]. However, research in this area is still in infancy in Ethiopia. To our knowledge, there is no data on knowledge, attitude, and practice of GS among pharmacy professionals in Jimma town. Therefore, the present study was aim to assess knowledge, attitude and practice among pharmacy professionals toward generic substitution in community drug retail outlets in Jimma town, Southwest Ethiopia**.**

## Methods

### Study area and period

The study was done in Jimma town. The town is in Oromiya regional state and is located about 352 km away in the southwest direction from Addis Ababa, the capital city of Ethiopia. According to the data from the Jimma Zone Health Bureau, there are five public health centers, one medical center, and two general hospitals, 32 drug stores and 26 community pharmacies at the time of data collection. The study was conducted from August 15 to 30, 2021.

### Study design

A descriptive cross-sectional study was carried out among all pharmacy professionals working in community drug retail outlets in Jimma town.

### Study participants and sampling procedure

The study participants were pharmacy professionals working in community drug retail outlets in Jimma town. Since there are limited number of community drug retail outlets in Jimma town, all volunteered pharmacy professionals were included in this study. Pharmacy professionals unwilling to participate were excluded from the study. Participation in the study was merely based on wllingness of the partcipants without any incentive.

### Data collection tools and techniques

A questionnaire was adopted from previously conducted researches with some modifications to suit the local context [2, 6, 11, 21, 22]. It has four sections: the first part contained socio-demographic information and the professional characteristics of study participants. The second part contained questions to test knowledge using a 3-point response format consisting of “Yes,” “No” and “I am not sure. The third section entailed statements to explore attitude toward GS for brand medicines using the 5-point Likert scale where 1 represents strong agreement and 5 represent strong disagreement. In the fourth section, a question related to the practice of generic medicines substitution was examined as never, seldom, sometimes, often, and always with scores ranging from 0 to 4. The questionnaire was a self-administered type. And all pharmacy professionals working in community drug retail outlets during the data collection period took part in the study. The questionnaire was physically disseminated. Some of the participants were completed the questionnaire immediately. Those who were volunteer to fill but busy at the time of questionnaire distribution received the questionnaire and filled. Then, they were communicated via phone and the questionnaire was recollected with appointment.

### Data processing and analysis

The data were analyzed using Statistical Package for Social Sciences version 22 (SPSS22). Frequencies and percentages of responses were produced for each answer in the questionnaire. Normality of data was tested using Kolmogorov-Smirnov test. The influences of respondents’ professional and demographic factors on knowledge, attitude, and practice were tested using the Mann–Whitney U and Kruskal–Wallis tests as appropriate. *P* ≤ 0.05 was considered as statistically significant association. For knowledge, the score of “1″ was given to correct answers, “0″ for incorrect or not sure and summative score was calculated. The total scores range from 0 to 9. The higher scores than over all mean score indicate more knowledge about GS. Similarly, all individual answers related to attitude and practice questions were computed to obtain the overall scores and means. The mean score was used to divide the participants into two groups. Respondents who scored greater or equal to the mean for attitude related questions were considered as having a positive attitude toward GS. And participants who scored greater or equal the mean for the practice question were considered as if they were practicing GS.

### Ethical approval process

The study protocol was reviewed and approved by Institutional Review Board of Institute of Health, Jimma University. Written ethical permission to conduct the study was requested from the Institutional Review Board of Institute of Health and permission from the concerned authorities was taken before the study commenced.

### Operational definition

#### Community drug retail outlets

It refers to all private pharmacies and drug stores in Jimma town.

#### Knowledgeable

If participants scored ≥ mean score for knowledge questions. Otherwise, they are considered Not- Knowledgeable.

#### Positive attitude

If participants scored ≥ mean score for attitude questions. Otherwise, they are considered to have a negative attitude.

#### Practice

If participants scored ≥ mean score for the practice questions were considered as they were practicing generic substitution.

#### Pharmacy professional

Refers to pharmacist and druggist in the context of this study.

## Results

### Socio-demographic characteristics

Out of 130 questioners distributed over 58 community drug retail outlets (DRO), 106 of the participants gave their responses giving a response rate of 81.5%. Of 106 participants, 67 (63.2%) were males. The highest percent (*n* = 72; 67.9%) of pharmacy professionals were between ages of 25–35-year-old. More than half (*n* = 61; 57.5%) of the participants had a bachelor’s degree (Table 1).

**Table 1:** Socio-demographic characteristics of pharmacy professionals (*n* = 106)

Variables	Category	N (%)
Age	25–35	72 (67.9)
	36–45	22 (20.8)
	≥46	12 (11.3)
Sex	Male	67 (63.2)
	Female	39 (36.8)
Educational level	Degree	61 (57.5)
	Diploma	45 (42.5)
Year of experience	1–5	70 (66)
	6–10	21 (19.8)
	≥11	15 (14.2)
Employment position	Employee	66 (62.3)
	Owner	32 (30.2)
	Co-owner	8 (7.5)

### Pharmacy professionals’ knowledge about generic substitution

Of the total nine knowledge items examined, the overall mean knowledge score for correctly answered questions was 5.75 ± 1.79. Accordingly, only 32 respondents (30.2%) scored mean or above mean and were considered knowledgeable about generic medicine and GS. Whereas 74 (69.8%) of respondents unexpectedly scored below the mean and were considered not knowledgeable (Table 2).

**Table 2:** Pharmacy professionals’ knowledge of generic medicines substitution (*n* = 106)

Knowledge Statements	Responses, n (%)		
	Right	Wrong	Not sure
1. A generic medicine is bioequivalent to brand medicine	87 (82.1)	15 (14.2)	4 (3.8)
2. Generic and brand medicines contain the same amount of active ingredients	86 (81.1)	18 (17.0)	2 (1.9)
3. Generic medicine only be marketed after the expiry date of brand medicine	61 (57.5)	32 (30.2)	13 (12.3)
4. Generic and brand medicines must be in the same dosage form	91 (85.8)	10 (9.4)	5 (4.7)
5. Generic medicine can be a substitute of brand medicine	34 (32.1)	68 (64.2)	4 (3.8)
6. Generic medicine is used at the same dose (s) to treat the same disease (s) as the brand medicine	75 (70.8)	15 (14.2)	16 (15.1)
7. Pharmacists are legally empowered to dispense generic medicine in place of prescribed brand medicine	81 (76.4)	17 (16)	8 (7.5)
8. Community pharmacists in Ethiopia have the right to perform generic substitution	60 (56.6)	32 (30.2)	14 (13.2)
9. Substitution of medicines with narrow therapeutic index is inappropriate	35 (33)	53 (50)	18 (17.0)
**Knowledge mean score (mean ± SD)**	**5.75 ± 1.79**	**NA**	**NA**

*NA* Not applicable, *SD* Standard deviation

The highest percent of the respondents (*n* = 91; 85.8%) correctly answered that generic and brand medicines must be in the same dosage form. Moreover, about eight 2 % (*n* = 87; 82.1%) of participant pharmacy professionals correctly identified that generic medicines are bioequivalent to brand medicines. Similarly, 86 (81.1%) respondents correctly replied to the statement ‘generic and brand medicines contain the same amount of active ingredients.” However, 53 (50%) pharmacy professionals wrongly understood that GS of medicines with a narrow therapeutic index is appropriate. Furthermore, majority (*n* = 68, 64.2%) of the participants wrongly responded generic medicine is not a substitute of brand medicine.

### Attitude of pharmacy professionals’ towards generic medicines substitution

The respondent’s attitude was assessed using eight attitudinal questions. The overall mean scores of participants’ attitudes toward GS were found to be 3.59 ± 0.46. Accordingly, 54 (50.9%) respondents scored mean or above the mean and were considered positive attitudes toward generic medicine and GS. From Table 3, an enormous portion (*n* = 63; 59.4%) of the participants disagreed or strongly disagreed that generic medicines are less effective than brand medicines. The study also indicated that 75 respondents (accounting for 70.7%) from 106 pharmacy professionals perceived that generic medicines are not lower quality than brand drugs. However, a majority (*n* = 81; 76.4%) of the respondents agreed therapeutic failure is a serious problem with most GS. Regarding the need for standard guidelines on GS, more than 90% of the pharmacy professionals in this study believed that a standard guideline is required for both prescribers and pharmacy professionals on the GS process (Table 3).

**Table 3:** Pharmacy professionals’ attitude toward generic medicines substitution (*n* = 106)

Attitude statements	Level of agreement, n (%)				
	1	2	3	4	5
1. As generic medicines are less effective than brand medicines substitution is not recommended	11 (10.4)	14 (13.2)	18 (17)	21 (19.8)	42 (39.6)
2. As generic medicines are of lower quality than brand medicines substitution is not recommended	14 (13.2)	17 (16)	–	35 (33)	40 (37.7)
3. I support generic substitution in all cases where generic alternatives are available	31 (29.2)	34 (32.1)	21 (19.8)	11 (10.4)	9 (8.5)
4. Generic substitution is only recommended for medicines with a high safety margin	44 (41.5)	41 (38.7)	11 (10.4)	5 (4.7)	5 (4.7)
5. I do generic substitution as I believe generic drugs and brands are bioequivalent	33 (31.1)	46 (43.4)	7 (6.6)	10 (9.4)	10 (9.4)
6. Therapeutic failure is a serious problem with most generic medicines substitution	34 (32.1)	47 (44.3)	7 (6.6)	14 (13.2)	4(3.8)
7. Community pharmacists should be allowed to perform generic substitution without consulting the prescribing physician	50 (47.2)	30 (28.3)	18 (17)	5 (4.7)	3(2.8)
8. There is a need for standard guidelines for prescribers and pharmacy professionals on generic medicine substitution process	69 (65.1)	31 (29.2)	2 (1.9)	4 (3.8)	–

1 = strongly agree, 2 = agree, 3 = neutral, 4 = disagree, 5 = strongly disagree

### Practice of pharmacy professionals towards the generic medicines substitution

The overall mean score of participants’ practice toward GS was 2.13 ± 0.64. Accordingly, 52 (49.1%) of respondents scored mean or above the mean and considered they were practicing GS. Twenty-two respondents (accounting for 20.8%) never choose generic medicines for self-treatment. On the other hand, about 90% (either seldom, sometimes, often, or always) of pharmacy professionals cited they dispensed equivalent generic medicine when brand medicine is available (Table 4).

**Table 4:** Practice of pharmacy professionals’ towards the generic substitution (*n* = 106)

Variable	N (%)				
	0	1	2	3	4
1. I choose generic drugs for self-treatment	22(20.8)	29 (27.4)	35 (33.0)	4(3.8)	16(15.1)
2. I dispense generic drug when brand medicine is prescribed	10(9.4)	28 (26.4)	36 (34.0)	24 (22.6)	8 (7.5)
3. I advise my clients about using of generic medicine	3(2.8)	7(6.6)	48 (45.3)	44 (41.5)	4 (3.8)
4. I encourage generic medicine substitution by prescribers	0(0)	27 (25.5)	16 (15.1)	39 (36.8)	24 (22.6)

0 = never, 2 = seldom, 3 = sometimes, 4 = often, 5 = always

### Influence of socio-demographic and professional characteristic of participants’ on their KAP towards generic substitution

Due to the non-normality of the data, the Mann–Whitney U and Kruskal–Wallis non-parametric tests were used. The influences of socio-demographic characteristics on mean scores were tested and only year of experience had a significant effect on the knowledge (X^2^ = 9.14, *p* = 0.01) and practice mean scores (X^2^ = 4.71, *p* = 0.03). The Mann–Whitney U test demonstrated a significant difference between less experienced pharmacy professionals compared to more experienced (1–5 years versus ≥11 years, U = 322.5, Z = **−** 2.895, *p* = 0.004). However, there was no statistically significant association between respondents’ socio-demographic characteristics and their attitude with regard to GS. The year of experience still significantly affected mean practice score (U = 535.5, Z = -2.170, *p* = 0.03). Accordingly, pharmacy professionals who had 6–10 years of experience had been practicing GS compared to those who had work experience of 1–5 years (Table 5).

**Table 5:** The influence of demographic and professional characteristics of participants’ on KAP of generic substitution

Socio-demographic variables		Knowledge		Attitude		Practice	
		Mean rank	Test statistic	Mean rank	Test statistic	Mean rank	Test statistic
Sex^a^	Male	54.47	U = 1241.5 Z = −0.54 *P* = 0.59	51.02	U = 1140.5 Z = −1.26 *P* = 0.21	52.60	U = 1246.5 Z = −0.45 *P* = 0.65
	Female	51.83		57.76		55.04	
Age^a,b^	25–35	51.83	X^2^ = 1.88,	55.27	X^2^ = 4.13	51.53	X^2^ = 3.4
	36–45	59.86	Df = 2,	56.79	Df = 2	62.64	Df = 2
	≥46	51.83	*P* = 0.39	40.63	*P* = 0.13	48.58	*P* = 0.18
Educational level^a^	Degree	55.37	U = 1288.5 Z = -0.66, P = 0.5	53.59	U = 1368.5 Z = -0.030 *P* = 0.98	51.23	U = 1270.5 Z = -0.753, *P* = 0.45
	Diploma	52.12		53.43		55.17	
Year of experience^a,b^	1–5	49.06	X^2^ = 9.14	56.68	X^2^ = 4.49	49.97	X^2^ = 4.76
	6–10	56.88	Df = 2	50.59	Df = 2	54.77	Df = 2
	≥11	69.5	**P = 0.01**	39.75	*P* = 0.106	64.36	**P = 0.03**
Employment position^a,b^	Employee	56.25	X^2^ = 1.88	49.69	X^2^ = 1.19	51.34	X^2^ = 1.12
	Owner	43.00	Df = 2	59.63	Df = 2	46.38	Df = 2
	Co-owner	53.44	P = 0.39	54.61	*P* = 0.551	55.41	*P* = 0.57

*Df* Degree of freedom, ^a^Mann-Whitney U tests, ^b^Kruskal-Wallis tests

## Discussion

Pharmacy professions’ knowledge, attitude, and practice (KAP) towards generic medicine substitution might affect the choice and use of therapeutic agents [19].

Concerning respondents’ knowledge of generic medicine substitution, the current study indicated, 82.1% of the participants correctly identified generic medicine as bioequivalent to brand medicine. This finding is higher than a similar study conducted in Mekelle town, Northern Ethiopia (52.9%) [20], Harar city, Eastern Ethiopia (67.6%) [9], New Zealand (70%) [14], and Qatar (38%) [2] but comparable with the result of the studies conducted in Addis Ababa, Ethiopia (77.1%) [13] and Palestine (76.5%) [1]. The present study also revealed that the majority of the study participants (*n* = 86; 81.1%) cited generic and brand medicines containing the same amount of active ingredients. This finding is in parallel with the result of studies done in Harar town (73%) [9], Mekelle town (76.1%) [20], and Addis Ababa (89%) [13]. Moreover, from the total participants, 85.8% of them declared that generic medicine must be in the same dosage form as the brand medicine. This is in line with the result of a study from Addis Ababa (84.4%) [13]. Nevertheless, it is higher than the report of study in Harar town (59.4%) [9] and Mekelle town (64.3%) [20]. This difference might be due to the difference in the level of qualification of the participants. In our study, the majority of the participants were bachelor’s degree holders.

According to our study, the majority (56.6%) of pharmacy professionals know that pharmacists in Ethiopia have the right to perform GS. This finding is in line with the report of a study in Bahir Dar (53.2%) [19] but lower than the finding of other previous studies from Harar town (67.5%) [9] and Addis Ababa (69.6% [13]. The discrepancy might arise from rigorous regulations and better access to such information concerning the brand and generic medicines in the case of a study done in Addis Ababa. In addition, nearly one-third (33%) of the respondents were wrongly cited that substitution of medicines with a narrow therapeutic index is appropriate. This finding is almost similar to the finding of a study done in Addis Ababa (29.1%) [13] but it is lower than the finding of a similar study in Harar town (23%) [9]. The variation is perhaps attributed to the difference in sample size.

Pharmacy professionals play a significant role in informing patients and other healthcare practitioners about generic medicines and GS [14] However, in our study, only about 30% of the pharmacy professionals working in community drug retail outlets were considered knowledgeable about generic medicine and GS. This response suggests the need for interventions. Though the reasons for the poor knowledge having positive attitude need further investigation to uncover underlining factors, absence of pilot study to pretest the questionnaires which may induce potential biases to the finding of the study was appreciated.

Regarding attitude, the overall pharmacy professionals’ attitudes on generic medicines substitution in our study were similar to a former study in Palestine [1] and Qatar [2]. This idea was revealed by 61.3% of the respondents who supported GS in all cases where generic alternatives are available. The finding is lower than the report from Qatar [2] but higher than the reports of the previous studies such as Bahir Dar town (51.1%) [19], Addis Ababa (48.3%) [13], Harar town (48.6%) [9] and Mekelle town (39.1%) [20]. This suggests that the pharmacy practitioner in our study had a positive attitude toward GS. Moreover, 75.5% of the participants declared that community pharmacists should be allowed to perform GS without consulting prescribing physicians. This finding is much higher than the previous studies report in Mekele town (50.5%) [20], Harar town (51%) [9] and Addis Ababa (66.2%) [13]. But it is lower than the report from Qatar [2].

Nevertheless, the current study indicated that about 23% of respondents thought generic medicines are less effective as opposed to their counterpart brand medicines. This value is comparable with the result of the studies from Bahir Dar (20.7%) [19]. But is less than the results reported from Poland (31%) [11], Addis Ababa (37.6%) [13], Mekelle town (34.4%) [20] and Harar town (48.5%) [9]. This suggests that our study found a greater score. This could be partly due to the increment of information about generic medicine with time. On the other hand, around 30% (agree = 16%, strongly agree = 13.2%) of the participants declared substitution of generic medicines is not recommended because generic medicines are of lower quality. This finding is almost analogous with the report of a study in Addis Ababa [13] where 33.7% of pharmacy professionals viewed generic medicine as of lower quality compared to brand medicines. This is in contrast to the finding of a study done in Harar town [9], Mekelle town [20] and New Zealand [14], where more than half (56.8, 51.7, and 65%, respectively) of the respondents perceived brand medicines as of higher quality.

Practice illustrates the dispensing of generic substitutes when brand medicines are prescribed to patients or preference of generic medicines for self-treatment. According to our study, nearly half (49.1%) of pharmacy professionals who participated in this study had practiced GS.

Concerning the influence of socio-demographic and professional characteristic of participants’ on their KAP towards GS, our study revealed that participants’ work experience had a significant effect on the knowledge score (U = 322.5, Z = − 2.895, *p* = 0.004). Pharmacy professionals with more years of practice scored higher on the knowledge of generic medicines than less experienced professionals. A similar finding was reported in studies conducted in Harar town [9], Addis Ababa [13], and Bahir Dar town [19]. The reason might be work experience enhance knowledge of pharmaceutical science and regulations regarding brand and generic medicine. However, a study from Qatar [2] reported there was no statistically significant difference (*p* > 0.05) between more experienced and less experienced pharmacy professionals on mean knowledge score. The other socio-demographic and work profiles were not significantly associated with the knowledge level of respondents.

The Mann–Whitney U and Kruskal–Wallis non-parametric tests revealed there was no significant association (*P* > 0.05) between the socio-demographic characteristics and the respondents’ attitude towards GS. There was no statistically significant association between respondents’ socio-demographic characteristics and their attitude with regard to GS. Work experience had also a significant effect on GS practice among the other socio-demographic characteristics. A study conducted in Mekelle town [20] reported that employment position (owner versus employee) had a statistically significant association with practice GS. It showed owner/co-owner had less tendency to perform GS than the employee. This discrepancy might due to the difference in interest as those business holders might have great interest toward their profit in the pharmaceutical market. But in our study, there was no significant association between employment position and GS practice. Generally, as the means score of knowledge, attitude and practice was used in the analysis, each knowledge, attitude and practice item’s effect was not studied. Moreover, as the mean score is an aggregate result, this might have underestimated the knowledge level of the respondents.

### Limitation of the study

Since a self-administered questionnaire was used, the response bias is likely and the associated self-reporting biases among the respondents are recognized. The presence of more than one pharmacy professionals per facility has a potential to contaminate the response. In addition, due to the relatively small sample size, and single site, the generalization of the results is limited as the sample of pharmacy professionals was taken from Jimma town alone which may not be representative of all pharmacy professionals’ in Jimma town and Ethiopia.

## Conclusions

Our study found that pharmacy professionals working in community drug retail outlets in Jimma town had lack of knowledge about generic substitution. Conversely, an enormous amount of participants had positive attitude toward generic substitution and nearly half of them had practiced generic substitution. The year of experience had a significant effect on knowledge and practice of generic substitution. Despite, there is still a concern of efficacy and quality of generic medicines among some of pharmacy professionals. Thus, training program is needed to ensure thorough understanding of efficacy and quality of generic medicines and promote generic substitution.

## Data Availability

The data used to support the findings of this study are available from the corresponding author upon reasonable request.

## Abbreviations

DRO: Community drug retail outlets
GS: Generic substitution
